# Increased Working From Home in Vocational Counseling Psychologists During COVID-19: Associated Change in Productivity and Job Satisfaction

**DOI:** 10.3389/fpsyg.2021.750127

**Published:** 2021-12-03

**Authors:** Andrea Zürcher, Sibylle Galliker, Nicola Jacobshagen, Peter Lüscher Mathieu, Andrea Eller, Achim Elfering

**Affiliations:** ^1^Master School of Advanced Studies in Psychology of Career Counselling and Human Resources Management (CCHRM), University of Bern, Bern, Switzerland; ^2^Department of Clinical Psychology and Psychotherapy, University of Bern, Bern, Switzerland; ^3^Department of Work and Organizational Psychology, University of Bern, Bern, Switzerland; ^4^National Centre of Competence in Research, Affective Sciences, University of Geneva, CISA, Geneva, Switzerland

**Keywords:** work from home (WFH), telework, remote work, vocational counseling, COVID-19 pandemic, productivity, job satisfaction

## Abstract

During the coronavirus disease 2019 (COVID-19) pandemic, vocational counselors in Switzerland more frequently worked from home (WFH) and less frequently worked on-site. The aim of this study was to assess how WFH corresponds with indicators of job performance and occupational wellbeing. More specifically, the current questionnaire study analyzed the increase in WFH, self-reported productivity, distractibility in WFH, current job satisfaction, work-life balance in WFH, and feeling of loneliness. Findings showed that the increase in WFH in vocational counseling psychologists during the COVID-19 pandemic was associated with an increase in productivity and job satisfaction and with lower distractibility in WFH compared to work on-site. However, more frequent WFH was not significantly associated with improved work-life balance during the COVID-19 pandemic. Vocational counselors who shared the office on-site with many colleagues experienced higher feeling of loneliness during WFH. Vocational counselors regarded the condition of WFH as productive and satisfying while work-life balance did not improve. The discussion sheds light on the potential WFH-related increase of boundary management demands.

## Introduction

The coronavirus disease 2019 (COVID-19) pandemic changed the work of many employees (Galliker et al., 2021a), on top of persisting occupational change toward more flexible work places and work times, as well as shorter working hours (Kamerāde et al., 2019; Balderson et al., 2020).

The same applied to vocational counselors, whose work environment changed significantly. Working from home (WFH) increased largely (Galliker et al., 2021a). Before COVID-19, most vocational counseling happened in person. During the COVID-19 pandemic, online counseling was introduced, which can be used on-site as well as from home.

Even though WFH before COVID-19 was not very common in vocational counselors, WFH has a long history and can have different forms, depending on the employment status and permanent vs. occasional working from home. Recently, the International Labour Organization distinguished home-based workers, homeworkers, and teleworkers (International Labour Organization, 2021). Based on this scheme, vocational counselors can be classified as permanently employed teleworkers who work at home on an occasional basis (International Labour Organization, 2021). Thus, telework is characterized by an increased use of information and communication technologies by employees (ICTs; Aborg et al., 2002; Messenger and Gschwind, 2016; International Labour Organization, 2021).

While working conditions in office have been studied extensively, knowledge about the working conditions at home and their impact on the occupational health and productivity of an employee is limited. In their meta-analysis, Gajendran and Harrison (2007) reported positive associations between telecommuting and mental health (telecommuting must not, but is often done at home). Grant et al. (2013) conducted in-depth interviews with 11 experienced workers who did WFH. Workers reported positive as well as adverse impacts of WFH on wellbeing, with the latter due to overworking and a lack of time for recuperation. A review of 12 studies highlights the health risks that arise from musculoskeletal complaints when the workplace design is inadequate in WFH (Wütschert et al., in press). A recent longitudinal study, however, did not find an increase in musculoskeletal pain in Swiss office workers who worked from home full time after the COVID-19 pandemic outbreak, although ergonomic conditions were worse at home (Aegerter et al., 2021). Moreover, no decrease in presenteeism was observed (Aegerter et al., 2021). Another recent large longitudinal study from February 2020 to February 2021, which covered the COVID-19 and lockdown-related increase in WFH, also found an increase in indicators of health and wellbeing and a decrease in presenteeism (Galliker et al., 2021a). Hence, despite mixed effects of WFH on employee health, more consistent evidence exists on positive effects of WFH on job performance and job satisfaction (e.g., Kröll and Nüesch, 2019; Galliker et al., 2021a).

For instance, Bloom et al. (2015) randomly assigned call center employees to either a WFH group or an on-site work group for 9 months. WFH led to a 13% performance increase. This improvement came mainly from a 9% increase in the number of minutes they worked during their shifts (e.g., by taking fewer breaks) and 4% originated from working faster (more calls per minute, attributed to a quieter and more convenient working environment). In accordance with the rather consistent findings on improved performance in WFH, we expect more frequent WFH to be associated with higher self-reported productivity (H1).

Nevertheless, performance in WFH might depend on working conditions in WFH. Even when it can be expected that certain ergonomic working conditions are worse at home compared to on-site work, other ergonomic conditions, such as distractibility, were rarely addressed in WFH so far. Wegner et al. (2011) found that teleworkers showed higher vigilance and more distinct inner calm when they worked from home than when they worked in an office. Since inner calm and higher vigilance both overlap with lower distractibility, we expect lower distractibility to be a performance-related advantage of WFH. For most vocational counselors, we expect distractibility in WFH to be lower than during work on-site. Therefore, we expect more frequent WFH to be related to less distractibility at home compared to on-site work (H2).

Evidence for higher job satisfaction in WFH is rather consistent (e.g., Kröll and Nüesch, 2019). Bloom et al. (2015) found not only productivity but also job satisfaction to increase by WFH. In addition to higher productivity and lower distractibility in WFH, we expect more frequent WFH to correspond with higher job satisfaction (H3).

Various studies have shown that employees who have flexible work arrangements, including WFH, experience less work-family conflict and less family-work conflict (Hill et al., 2003; Byron, 2005; Kelly et al., 2008; Joyce et al., 2010; Solís, 2016). Conflicts between work and family life reflect a low work-family balance that is defined as “the extent to which an individual is equally engaged in—and equally satisfied with—his or her work role and family role” (Greenhaus et al., 2003, p. 513). Using a broader conceptual approach that does not only refer to family life as the major domain of life outside work (Guest, 2002), Grant et al. (2013) found that WFH made work life and non-work life more compatible and therefore improved work-non-work-life balance. Hence, more frequent WFH should be associated with a better work-life balance (H4).

There is evidence that commuting times may threaten work-life balance (Bai et al., 2021). As a positive consequence of WFH, commuting days per week decreased between February 2020 and February 2021 in Switzerland (Galliker et al., 2021a). In 2019, the average commuting time to work in Switzerland was 29.5 min one way (Bundesamt für Statistik [BFS], 2021). The duration of commuting is significantly associated with lower wellbeing next to many task demands, job resources, as well as private demands and resources (Elfering et al., 2020; Gerpott et al., 2021). During COVID-19 pandemic, longer commuting times may also be perceived as a higher risk of infection. Hence, more frequent WFH should be associated with a better work-life balance when (the thus saved) commuting time is longer (H5).

The study of loneliness at the workplace is a relatively new research field (Wright and Silard, 2021).

Wood et al. (2021) found that loneliness—besides the ability to detach from work—is the crucial factor in the changing level of wellbeing in WFH. In the experimental study of Bloom et al. (2015), 50% of WFH group participants wanted to switch back to on-site work. “Loneliness was the single biggest reason.” Bloom explained in an interview with *The Guardian* (Usborne, 2020). During the COVID-19 pandemic, physical isolation during work may have resulted in increased social isolation because of reduced work contacts and predominant virtual communication (Lengen et al., 2021). Therefore, WFH may be associated with feeling of isolation from colleagues when vocational counselors—before the COVID-19 pandemic—were used to sharing the office with one or more colleagues and now do WFH. Hence, the current study expects feeling of loneliness during WFH to correspond positively with the number of office coworkers before the COVID-19 pandemic (H6).

### Hypotheses

**FIGURE 1:**
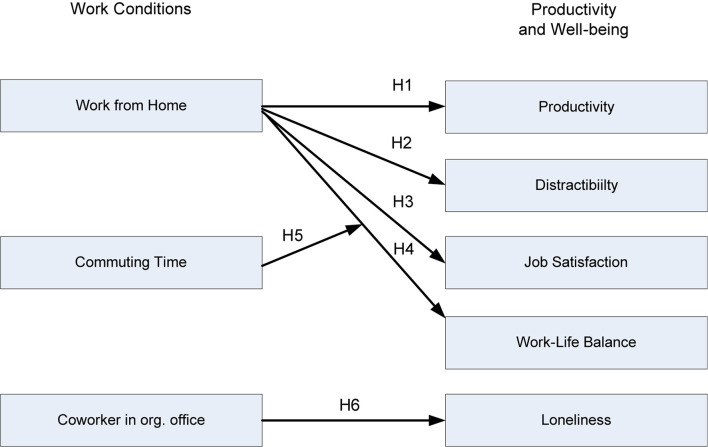
Study hypotheses on working conditions during the coronavirus disease 2019 (COVID-19) pandemic and self-reported productivity, distractibility, and indicators of wellbeing.

The study hypotheses are shown in Figure 1.

**Hypothesis 1:** More frequent WFH is associated with a higher work productivity in WFH compared to work on-site.
**Hypothesis 2:** More frequent WFH corresponds to lower distractibility in WFH compared to work on-site.
**Hypothesis 3:** More frequent WFH is associated with higher job satisfaction.
**Hypothesis 4:** More frequent WFH is associated with a better work-life balance during the COVID-19 pandemic.
**Hypothesis 5:** More frequent WFH is more strongly associated with better work-life balance during the COVID-19 pandemic when commuting time is longer.
**Hypothesis 6:** Vocational counselors who share the office on-site with many colleagues experience higher feeling of loneliness when they are working from home.

## Materials and Methods

### Data Collection

The participants were recruited through various channels. The first author (AZ) contacted most of the organizations in German-speaking Switzerland directly, including the heads of the offices, with the invitation to her survey and request for forwarding. The same applied to the academic advising centers of the universities and universities of applied sciences in German-speaking Switzerland. The participants could indicate if they were interested in the survey results. A total of 266 vocational counselors were solicited. This resulted in a response rate of 89%. It is not known whether participants could complete the survey during working hours, except for one public career counseling center that explicitly allowed this. The survey took place from early November 2020 to early December 2020. The participants were primarily asked about their current work situation in November and December 2020. It is important to note that on October 18, 2020, the Swiss Federal Council increased protective measures against the then sharply rising infection rates. More specifically, it recommended to work from home whenever possible (Federal Council media release of October 18, 2020).

The duration of the survey was approximately 15 min. All data were collected completely anonymously. The study participants were informed of the content of the study and its voluntary participation. The study language was German. The study was approved by the ethics committee of the University of Bern, Switzerland (12.01.21, Ethics No. 2021-01-00001).

### Sample Characteristics

The sample consisted of 238 vocational, academic, and career counselors from German-speaking Switzerland. The participants included career counselors from occupational rehabilitation offices, cantonal vocational counseling offices, academic career counseling offices at universities, and private career counseling. In addition, some participants worked in related fields, such as internal career counseling in large companies or in institutions for bridge offers. This was an accrual sample, as it was not stratified and randomly selected.

The majority of participants were females (68.9%), married (82.4%), had a university/technical college degree (95.8%), and worked at a public career guidance office (61.8%). Specifically, more public career counselors participated in the survey than rehabilitation career counselors (21.0%). It is important to note that there are more public than rehabilitation vocational counselors throughout Switzerland. Furthermore, the contact details of the public vocational guidance offices are freely available on the Internet and could be contacted directly. The participants worked an average of 75% and were 46.2 years old. They had an average of 10.6 years of professional experience and had been working for the same employer for 8.5 years. Compared to public career counselors, rehabilitation vocational counselors were slightly younger on average, had less professional experience (also the duration of employment was somewhat lower), and worked at least 50% (vs. 30% for the public counselors).

At the time of data collection, 69.3% of respondents worked from home. Before the COVID-19 pandemic, there were only 28.2% participants who reported to WFH. Equally, there were only a few respondents who solely worked from home. The majority worked at home up to 10 days per month (no WFH: 30.7%; half a day to 5 days/month: 41.3%; 6–10 days/month: 15.6%, more than 10 days/month: 12.4%). Furthermore, the majority (91.5%) of participants who did WFH stated that they had an undisturbed work environment at home. The reasons why participants did not work from home were assessed in an open question and coded by a blind rater. Most respondents reported that they preferred to meet clients in person (25 out of 73) or that clients preferred a personal contact (19 out of 73). Sixteen participants explained that their office on-site was better equipped than their office at home. Finally, 13 participants reported their supervisor wanted them to work on-site.

### Measures

#### Predictor Variables

*Working from home (WFH)* was assessed by asking subjects whether they worked from home (“Do you work from home?”) with “Yes” and “No” as response options. If participants answered “no,” they were asked to explain why in an open question. Participants were then asked whether they had already worked at home before the first corona-related lockdown (March 2020–June 2020) and, if so, how many days per month on average. Furthermore, they were asked whether they were *currently* working at home and, if so, how many days per month on average. Thus, “currently” referred to the time of data collection in November/December 2020. The questions used were adapted from the WFH questions used by Galliker et al. (2021a).

*Commuting time* was assessed by asking participants the duration of one-way commute from home to work, where 1 = less than an hour, 2 = about 1 h, and 3 = between 1 and 2 h.

*Coworker in the on-site office* was assessed by asking participants with how many coworkers they shared the on-site office.

#### Outcome Variables

Some outcome measures asked participants to compare their current work situation with the situation before the corona-related lockdown (March–June 2020). They were explicitly not asked about WFH during the corona-induced lockdown, as many other factors would have influenced the results during this time (e.g., homeschooling of the children).

If not specified otherwise, response options were indicated on a five-point Likert response scale (1 = completely agree, 2 = disagree, 3 = undecided, 4 = agree, and 5 = completely agree).

*Productivity* addressed WFH productivity versus on-site productivity (“When working from home, I am more productive than on-site”). The item used was adapted from the WFH questionnaire developed by Aczel et al. (2021).

*Distractibility* was measured with the following item: “When working from home, I am less distracted than on-site.” The item was adapted from the WFH questionnaire developed by Aczel et al. (2021).

*Work-life balance.* The balance between work and non-work life was assessed by a single item adopted from Lonska et al. (2021): “Since the COVID-19 pandemic, work-life balance has improved.”

*Loneliness* during WFH was assessed with the following item: “Sometimes I feel lonely when working from home,” a question that was adopted from Golden et al. (2008).

*Job satisfaction.* The Kunin faces question (KFQ) was used to measure job satisfaction (Kunin, 1955). The KFQ is a single-item measure of overall satisfaction that focuses primarily on the affective component of job satisfaction compared with other scales (Elfering and Grebner, 2011; Elfering et al., 2016). The KFQ asked “How satisfied do you currently feel with your work?” This measure was assessed with seven smiley faces with written labels, ranging from a deep frown (1 = very unsatisfied) to a large smile (7 = very satisfied). Wanous et al. (1997) reported a good reliability and validity of the KFQ single-item measure of overall job satisfaction.

### Control Variables

Age, gender, relationship status, leadership function, part-time work, number of children, and work demands were included as control variables in the regression analyses.

*Age* was included as a control variable because work and private demands that have an impact on work-life balance change with age (Rantanen et al., 2012). Furthermore, job satisfaction has been shown to be positively related to age (Spector, 1997).

*Gender* (i.e., 1 = female and 2 = male) was included as a control variable because gender differences in self-rated work productivity and job satisfaction during the lockdown may arise (Feng and Savani, 2020). Women are expected to spend more time on domestic work and childcare than their male counterparts when working from home.

*Relationship status* was included as a control variable because being in a relationship can alleviate stress or buffer the stressor-strain association (Grandey and Cropanzano, 1999). It was operationalized as “in a relationship” or “single.”

*Leadership function (yes/no).* Working from home with leadership function does afford new remote leadership behavior (Parker et al., 2020; De Bloom and Keller, 2021a,b; Schall and Chen, 2021).

*Number of children living in the same household.* During the COVID-19 pandemic, new demands emerged with having children who also had to adopt to circumstances like home schooling (Harth and Mitte, 2020).

*Part-time work* can reduce work-life conflict (Roeters and Craig, 2014). Part-time work of an employed person was assessed in percentages of a full-time work equivalent (e.g., 60% of 100%; in Switzerland, 100% corresponds to 42 h of working time per week).

*Job demands* were included as control variables as working from home can introduce alterations in job demands (Sardeshmukh et al., 2012). Job demands were assessed using the scales quantitative work stress (e.g., “I have too much work”) and qualitative work stress (e.g., “At this work, there are things that are too complicated”) of the “Short Questionnaire for Work Analysis” (Prümper et al., 1995). The respective scales contain two items each. Response options were indicated on a five-point Likert response scale (“strongly disagree” to “strongly agree”). Cronbach’s alpha was 0.89 for quantitative work stress and 0.69 for qualitative work stress.

### Statistical Analyses

We used IBM SPSS Statistics 23, Armonk, NY, New York for all analyses. For the multiple regression analysis, we calculated linear regression models using the enter method. The multiple linear regression consisted of two steps: step 1 included control variables and step 2 included predictor variables [days WFH (# days/month), commuting time, and days WFH × commuting time or number of coworkers in the office on-site]. When examining hypothesis 5 with the interaction term, the predictor variables were centered.

We used an alpha level of 0.05, and the tests were two-tailed.

## Results

### Descriptive Results

The mean values of study variables are shown in Table 1. When asked whether productivity in WFH was higher than on-site, most respondents were undecided in this question. Slightly more respondents agreed than disagreed (12.2% completely disagreed, 18.5% disagreed, 31.5% were undecided, 29.4% agreed, and 8.4% completely agreed).

**TABLE 1:** Mean values and Pearson correlations between study variables.

	***M***	***SD***	**1**	**2**	**3**	**4**	**5**	**6**	**7**	**8**	**9**	**10**	**11**	**12**	**13**	**14**	**15**	**16**
1. Higher productivity in WFH	3.03	1.14	1															
2. Less distractibility in WFH	3.29	1.26	0.78***	1														
3. Higher loneliness in WFH	2.59	1.17	−0.16*	−0.15*	1													
4. Better work-life balance in WFH	4.28	0.87	0.01	0.09	−0.21**	1												
5. Job satisfaction	5.28	1.06	0.09	0.01	–0.09	0.18**	1											
6. Qualitative work demands	2.09	0.92	0.04	0.08	0.18**	−0.16*	−0.21***	1										
7. Quantitative work demands	3.06	0.96	0.18**	0.14*	0.09	−0.22***	−0.21**	0.45***	1									
8. WFH (# days/month)	4.02	4.83	0.31***	0.25***	0.14*	–0.04	0.11	0.15*	0.23***	1								
9. Coworker in org. office	1.07	1.65	0.23***	0.24***	0.18**	–0.02	0.03	0.17**	0.14*	0.41***	1							
10. Commuting time	1.41	0.66	0.14*	0.16*	–0.03	0.06	0.06	0.07	0.12	0.13*	0.01	1						
11. Age (years)	46.23	9.76	−0.15*	−0.18**	–0.09	0.14*	0.08	–0.05	−0.16*	−0.15*	−0.16*	–0.02	1					
12. Gender (1 = female, 2 = male)	1.31	0.46	−0.16*	−0.14*	–0.01	–0.02	0.01	0.11	0.04	–0.06	–0.05	0.04	0.11	1				
13. Partnership (0 = no, 1 = yes)	0.82	0.38	–0.03	–0.09	–0.10	0.04	0.04	0.01	–0.06	–0.05	0.01	0.09	0.08	0.07	1			
14. Leadership (0 = no, 1 = yes)	0.13	0.34	0.02	0.09	−0.17*	–0.03	0.10	–0.11	0.06	0.04	–0.06	–0.13	0.03	0.09	0.08	1		
15. Part–time work (%FTE)	75.18	13.86	0.06	0.13*	0.06	0.02	–0.11	0.06	0.19**	0.15*	0.12	−0.13*	–0.11	0.25***	−0.24***	0.22***	1	
16. Children	0.82	1.04	0.02	–0.08	–0.09	–0.06	0.21**	–0.04	0.01	–0.08	–0.04	–0.06	–0.01	0.10	0.27***	0.21***	−0.35***	1

*N* = 238; %FTE = percentage of full-time equivalent. *p < 0.05, **p < 0.01, ***p < 0.001.

The pattern of answers is more pronounced with respect to WFH when respondents were asked whether distraction in WFH was lower than on-site. The majority of respondents agreed that distractibility in WFH is lower than on-site work (11.8% completely disagreed, 16.9% disagreed, 19.0% were undecided, 35.4% agreed, and 16.9% completely agreed).

The mean value of current job satisfaction (5.28) reflects more than 80% of respondents who report being satisfied, very satisfied, or extremely satisfied with their current work (0.4% extremely unsatisfied, 1.9% very unsatisfied, 3.4% unsatisfied, 13.9% undecided, 32.4% satisfied, 41.2% very satisfied, and 7.1% completely satisfied).

Regarding the work-life balance, the pattern is in favor of WFH. More than three-quarters of respondents agreed that since the COVID-19 pandemic outbreak, their work-life balance has improved (no respondent completely disagreed, 4.6% disagreed, 13.5% were undecided, 30.8% agreed, and 51.1% completely agreed). Loneliness in WFH was denied by most respondents. Only a quarter of participants agreed that they sometimes feel lonely when working from home (21.1% completely disagreed, 29.1% disagreed, 23.6% were undecided, 21.5% agreed, and 4.6% completely agreed).

Pearson correlations of study variables are shown in Table 1. The correlation between productivity and distractibility is very high (*r* = 0.78, *p* < 0.001). Both higher productivity in WFH and lower distractibility in WFH are positively related with more frequent WFH (productivity: *r* = 0.31, *p* < 0.001; distractibility: *r* = 0.25, *p* < 0.001) and quantitative work demands (productivity: *r* = 0.18, *p* < 0.01; distractibility: *r* = 0.14, *p* < 0.05). Higher productivity in WFH and lower distractibility in WFH also corresponded with longer commuting times (productivity: *r* = 0.14; *p* < 0.05; distractibility: *r* = 0.16, *p* < 0.05). Job satisfaction showed negative associations with quantitative work demands (*r* = −0.21, *p* < 0.01) and qualitative work demands (*r* = −0.21, *p* < 0.001) but no significant associations with frequency of WFH. The work-life balance also showed negative associations with quantitative (*r* = −0.22, *p* < 0.001) and qualitative work demands (*r* = −0.16, *p* < 0.05) but no associations with frequency of WFH. Higher qualitative work demands correspond to higher feeling of loneliness in WFH (*r* = 0.18, *p* < 0.01). Sharing the on-site office with more colleagues is associated with higher feeling of loneliness in WFH (*r* = 0.18, *p* < 0.01). More frequent WFH was also slightly associated with higher feeling of loneliness (*r* = 0.14, *p* < 0.05).

### Hypotheses Testing

All hypotheses were examined with the multiple linear regression analysis. Informal examination of the data with histograms and scatterplots revealed no serious threats to underlying distributional assumptions of the residuals.

By testing the first hypothesis, a multiple regression was carried out to investigate whether WFH could significantly predict productivity above and beyond control variables. The results of the regression indicated that the model explained 14.1% of the variance and that the model was a significant predictor of productivity [Table 2; *F*(9,224) = 4.094, *p* < 0.001]. WFH contributed significantly to the model (β = 0.267, *p* < 0.001, variation explained by WFH = 6.5%; Δ*R^2^* = 0.065), corroborating hypothesis 1, which predicted that more frequent WFH was related to higher self-reported productivity in WFH compared to work on-site above and beyond control variables.

**TABLE 2:** Multiple linear regression analysis for work from home (WFH) predicting higher productivity in WFH compared to work on-site (H1).

**Variable**	***B***	***SE B***	**β**	***t***	***p***
Age (years)	–0.008	0.008	–0.066	–1.019	0.309
Gender (1 = female, 2 = male)	–0.384	0.167	−0.154*	–2.302	0.022
Partnership (0 = no, 1 = yes)	0.034	0.197	0.011	0.170	0.865
Leadership (0 = no, 1 = yes)	–0.060	0.233	–0.018	–0.259	0.796
Part-time work (%FTE)	0.005	0.006	0.061	0.786	0.433
Children	0.078	0.081	0.070	0.955	0.340
Qualitative work demands	–0.059	0.088	–0.048	–0.675	0.500
Quantitative work demands	0.146	0.087	0.122	1.683	0.094
WFH (# days/month)	0.063	0.015	0.267***	4.113	<0.001
Total *R*^2^			0.141***		<0.001
*F*(9,224) = 4.094					

*N* = 234; *p < 0.05, ***p < 0.001.

The second hypothesis examined whether more frequent WFH corresponds to lower distractibility in WFH compared to work on-site. Another multiple linear regression was calculated to predict distractibility based on WFH and control variables. A significant regression equation was found [Table 3; *F*(9,223) = 3.446, *p* < 0.001] with an *R* squared value of 0.122. The results of the multiple linear regression indicated that the model explained 12.2% of the variance of distractibility. Moreover, WFH was a significant predictor of distractibility (β = 0.185, *p* = 0.01; variation explained by WFH = 3.1%; Δ*R^2^* = 0.031). In line with the second hypothesis, more frequent WFH predicted less distractibility in WFH compared to work.

**TABLE 3:** Multiple linear regression analysis for work from home (WFH) predicting less distractibility in WFH compared to work on-site (H2).

**Variable**	***B***	***SE B***	**β**	***t***	***p***
Age (years)	–0.015	0.009	–0.117	–1.785	0.076
Gender (1 = female, 2 = male)	–0.392	0.187	−0.143*	–2.103	0.037
Partnership (0 = no, 1 = yes)	–0.106	0.220	–0.032	–0.483	0.630
Leadership (0 = no, 1 = yes)	0.363	0.260	0.096	1.396	0.164
Part–time work (%FTE)	0.007	0.007	0.075	0.960	0.338
Children	–0.047	0.091	–0.038	–0.520	0.603
Qualitative work demands	0.055	0.099	0.040	0.561	0.575
Quantitative work demands	0.061	0.097	0.046	0.632	0.528
WFH (# days/month)	0.049	0.017	0.185**	2.814	0.005
Total *R*^2^			0.122***		<0.001
*F*(9,223) = 3.446					

*N* = 233; *p < 0.05, **p < 0.01, ***p < 0.001.

The third hypothesis postulated more frequent WFH to be associated with higher job satisfaction. Table 4 shows the results of the corresponding multiple linear regression analysis where job satisfaction was predicted by WFH and control variables. The results of the regression illustrated that the multiple linear regression model explained 15.2% of the variance in job satisfaction as the dependent variable. The regression equation was found to be significant [Table 4; *F*(9,224) = 4.452, *p* < 0.001]. In compliance with hypothesis 3, WFH contributed significantly to the model (β = 0.215, *p* = 0.001; variation explained by WFH = 4.2%; Δ*R^2^* = 0.042).

**TABLE 4:** Multiple linear regression analysis for work from home (WFH) predicting job satisfaction (H3).

**Variable**	***B***	***SE B***	**β**	***t***	***P***
Age (years)	0.009	0.007	0.080	1.248	0.213
Gender (1 = female, 2 = male)	0.043	0.153	0.019	0.284	0.776
Partnership (0 = no, 1 = yes)	–0.113	0.181	–0.041	–0.625	0.533
Leadership (0 = no, 1 = yes)	0.142	0.213	0.045	0.667	0.505
Part-time work (%FTE)	–0.003	0.006	–0.039	–0.503	0.616
Children	0.217	0.075	0.211**	2.916	0.004
Qualitative work demands	–0.163	0.081	−0.142*	–2.019	0.045
Quantitative work demands	–0.198	0.080	−0.180*	–2.490	0.014
WFH (# days/month)	0.047	0.014	0.215**	3.330	0.001
Total *R*^2^			0.152***		<0.001
*F*(9,224) = 4.452					

*N* = 234; *p < 0.05, **p < 0.01, ***p < 0.001.

According to the fourth hypothesis, more frequent WFH is expected to significantly predict better work-life balance during the COVID-19 pandemic beyond control variables. Testing hypothesis 4 in a multiple repression analysis resulted in only 7.1% of the variance in work-life balance that could be explained by the regression equation [Table 5; *F*(9,223) = 1.907, *p* = 0.052]. WFH was not a significant predictor of work-life balance (β = 0.024, *p* = 0.726). Thus, hypothesis 4 was not supported.

**TABLE 5:** Multiple linear regression analysis for work from home (WFH) predicting improved work-life balance during COVID-19 pandemic (H4).

**Variable**	***B***	***SE B***	**β**	***t***	***p***
Age (years)	0.010	0.006	0.115	1.705	0.090
Gender (1 = female, 2 = male)	–0.074	0.132	–0.039	–0.561	0.576
Partnership (0 = no, 1 = yes)	0.113	0.156	0.050	0.728	0.468
Leadership (0 = no, 1 = yes)	–0.125	0.184	–0.048	–0.683	0.495
Part-time work (%FTE)	0.005	0.005	0.084	1.041	0.299
Children	–0.022	0.064	–0.026	–0.341	0.733
Qualitative work demands	–0.072	0.070	–0.076	–1.031	0.304
Quantitative work demands	–0.162	0.069	−0.179*	–2.357	0.019
WFH (# days/month)	0.004	0.012	0.024	0.351	0.726
Total *R*^2^			0.071		0.052
*F*(9,223) = 1.907					

*N* = 233; *p < 0.05.

The fifth hypothesis expected that more frequent WFH would be associated with better work-life balance during the COVID-19 pandemic when commuting time is longer. By testing the fifth hypothesis, a multiple linear regression was carried out that included control variables, WFH, commuting time and the interaction between WFH, and commuting time in a regression equation. Table 6 shows that the regression model explained only 7.7% of the variance in work-life balance as a dependent variable. The combination of independent variables in the regression equation did not achieve a significant prediction of work-life balance [Table 6; *F*(11,221) = 1.681, *p* = 0.079]. The interaction between WFH and commuting time did not significantly contribute to the regression model (β = −0.009, *p* = 0.894). Hence, hypothesis 5 was not confirmed.

**TABLE 6:** Multiple linear regression analysis for the interaction between work from home (WFH) and commuting time in predicting improved work-life balance during COVID-19 pandemic (H5).

**Variable**	***B***	***SE B***	**β**	***t***	***p***
Age (years)	0.003	0.002	0.122	1.803	0.073
Gender (1 = female, 2 = male)	–0.025	0.037	–0.047	–0.670	0.503
Partnership (0 = no, 1 = yes)	0.022	0.043	0.035	0.500	0.618
Leadership (0 = no, 1 = yes)	–0.043	0.051	–0.060	–0.842	0.401
Part-time work (%FTE)	0.002	0.001	0.110	1.321	0.188
Children	0.001	0.018	0.004	0.054	0.957
Qualitative work demands	–0.019	0.019	–0.073	–0.989	0.324
Quantitative work demands	–0.048	0.019	−0.190*	–2.477	0.014
WFH (# days/month)	0.001	0.003	0.021	0.308	0.758
commuting time	0.032	0.025	0.089	1.302	0.194
WFH × commuting time	–0.001	0.006	–0.009	–0.133	0.894
Total *R*^2^			0.077		0.079
*F*(11,221) = 1.681					

*N* = 233; *p < 0.05.

Finally, the sixth hypothesis postulated that vocational counselors, who shared the on-site office with many colleagues, should be more prone to feeling of loneliness in WFH. A test of the sixth hypothesis also relied on a multiple linear regression to examine whether the number of coworkers in the on-site office could significantly predict loneliness in WFH above and beyond control variables. The results of the multiple linear regression indicated that the regression model accounted for 8.9% of the variance in feeling of loneliness that were explained by the regression model [Table 7; *F*(9,223) = 2.416, *p* = 0.012]. In line with hypothesis 6, the number of coworkers in the on-site office contributed significantly to the multiple regression model (β = 0.145, *p* = 0.030; variation explained by coworkers in the on-site office = 2.0%; Δ*R^2^* = 0.020).

**TABLE 7:** Multiple linear regression analysis for coworker in the office on-site predicting loneliness in WFH (H6).

**Variable**	***B***	***SE B***	**β**	***t***	***p***
Age (years)	–0.005	0.008	–0.041	–0.609	0.543
Gender (1 = female, 2 = male)	0.038	0.175	0.015	0.219	0.827
Partnership (0 = no, 1 = yes)	–0.252	0.209	–0.082	–1.206	0.229
Leadership (0 = no, 1 = yes)	–0.488	0.244	−0.140*	–1.997	0.047
Part-time work (%FTE)	0.003	0.007	0.036	0.444	0.658
Children	0.005	0.085	0.005	0.061	0.952
Qualitative work demands	0.173	0.094	0.137	1.848	0.066
Quantitative work demands	0.004	0.091	0.004	0.047	0.962
Coworker in org. office	0.102	0.047	0.145*	2.187	0.030
Total *R*^2^			0.089*		0.012
*F*(9,223) = 2.416					

*N* = 233; *p < 0.05.

In summary, the results of regression analyses supported H1, H2, and H3: more frequent WFH was associated with higher self-reported productivity in WFH compared to work on-site (H1), lower distractibility during WFH compared to work on-site (H2), and higher job satisfaction (H3). Hypotheses 4 and 5, in contrast, were not confirmed: More frequent WFH was not associated with higher work-life balance (H4). Interestingly, the reduction in commuting times due to increased WFH during the COVID-19 pandemic was not found to be linked to a higher work-life balance (H5). However, the results confirmed hypothesis 6: a higher number of coworkers in the on-site office was related to higher feeling of loneliness during WFH (H6).

## Discussion

In this study, the results confirm that vocational counseling psychologists working from home (WFH) reported lower distractibility, higher productivity, and job satisfaction compared to working on-site. Furthermore, sharing on-site office with coworkers explains feeling of loneliness when working from home during COVID-19 confinement. Contrary to our expectations, WFH and reduction of commuting time do not explain work-life balance.

### Working From Home, Productivity, Lower Distractibility, and Job Satisfaction

The current study found higher self-reported productivity in WFH compared to work on-site. A similar result was recently reported in a large Swiss population study that included two measurement points: the baseline questionnaire was collected in February 2020, before the COVID-19 pandemic started, followed by the second questionnaire 1 year later in February 2021 (Galliker et al., 2021a). In this longitudinal study, health-related productivity loss was measured by WPAI (absenteeism and presenteeism) and monthly income. WFH was a significant predictor of productivity, next to job stressors and resources (Galliker et al., 2021a). Future studies should also disentangle different aspects of work productivity that might differently change in WFH (e.g., innovation in WFH; Kniffin et al., 2021). Moreover, it would be interesting to analyze objective measurements of productivity, e.g., the number of clients or the number of counseling sessions.

The frequency of WFH was significantly associated with lower distractibility in WFH compared to work on-site as proposed by H2. A lower distractibility in WFH compared to working on-site may point to more privacy during WFH. A recent study on the role of privacy in WFH showed a significant indirect effect of the level of privacy in WFH via cognitive irritation, indicating a lack of detachment from work issues, resulting in sleeping problems (Wütschert et al., 2021). Hence, distractibility in WFH in relation to work on-site is not only closely linked to productivity but—as a proxy to privacy in WFH and work on-site—also to recovery from work. As a lesson learned from the COVID-19 pandemic, the study of WFH-specific work conditions, including detachment from work, becomes an important goal in work psychology (Rudolph et al., 2021). Furthermore, knowledge about how to best detach in WFH is still scarce. Not surprisingly, some practical strategies from the literature and popular press address privacy in WFH, e.g., having a separate office with a door (Rudolph et al., 2021).

More frequent WFH was associated with higher job satisfaction. The expected findings confirm the evidence from meta-analyses on WFH and important job outcomes, including job satisfaction (Gajendran and Harrison, 2007; Allen et al., 2015). It is important to note that there seems to be some evidence for a non-linear association between the frequency of WFH and job satisfaction, postulating a decreasing job satisfaction for those who nearly always work from home. In the current study, however, the frequency of WFH was only low to moderate with the majority of participants who worked only between 1 and 5 days per month at home. Hence, the linear relationship between the frequency of WFH and job satisfaction in the current study might partly reflect a range restriction in WFH.

### Working From Home, the Commuting Time, and Work-Life Balance

Working from home is less restricted to normal office times than work on-site (Jostell and Hemlin, 2018). Therefore, WFH might promote online counseling at unusual work times (for instance, in the evening, after clients have finished their work). As a result, the worktime boundary between work and private life in WFH could gradually disappear. Thus, a potential dissolution of boundaries between work and personal life might result from WFH in vocational counselors. This might have a negative effect on work-life balance, which was originally found to be a positive consequence of WFH (Sinclair et al., 2020; Allen et al., 2021). Therefore, future studies should assess and test the usefulness of the so-called segmentation norms that protect from work-related technology use at home during non-work hours (Park et al., 2011).

Another preventive approach is to increase boundary control. Boundary control is a preventative measure against disappearing boundaries between work and private life in WFH. Boundary control is defined as the perception that an individual influences the transitions between work and family domains in WFH (e.g., the timing, frequency, and direction of transitions; Kossek and Lautsch, 2012). Future studies should test boundary control as a potential moderator of the association between frequency of WFH and work-life balance. The expectation would be that more frequent WFH is related to a better work-life balance but only when boundary control is sufficient. From the meta-analysis of Gajendran and Harrison (2007), researchers learned that WFH mostly includes a gain of autonomy in managing the interface between work and home (i.e., boundary control), but that gain may have been decreased in the latest decade by rising demands to be available 24/7 (Dettmers, 2017). Rudolph et al. (2021) claimed that research should focus on practical strategies to preserve role segmentation in WFH.

In the current study, commuting time and the amount of reduction in commuting times due to increased WFH during the COVID-19 pandemic were not significantly associated with work-life balance. One reason could be the range restriction in WFH: in the current study, the frequency of WFH was only low to moderate with the majority of participants working only between 1 and 5 days per month at home. Therefore, the reduction of commuting times due to increased WFH was not that essential for the majority of participants. Another reason might be that the role-segregating function of commuting might have outweighed the burden of time costs on private life. In WFH, an alternative “commute strategy,” such as walking around the block to mentally detach, should be evaluated (Rudolph et al., 2021). Clearly, the first major aim within boundary management is to ensure that WFH is *not* used as a form of childcare (Rudolph et al., 2021). Ideally, work design in WFH supports boundary management when employees proactively create conditions within work activities from home that foster enjoyment and challenge (Bakker and van Wingerden, 2021).

### Working From Home, Working Conditions, and Loneliness

The experience of social connectedness was found to be a health-related work resource in WFH (Oakman et al., 2020). A recent study on work conditions, performance, and wellbeing in WFH identified loneliness as an important remote work challenge (Wang et al., 2021). In the current study, feeling of loneliness in WFH was prevalent in one out of four participants. Vocational counselors who shared the on-site office with many coworkers were likely to report stronger feeling of loneliness while working from home (H6). Significant negative association between loneliness and the sheer amount of face-to-face interactions was found in former studies (Jin and Park, 2010). Even the greater decrease in daily face-to-face contacts could therefore cause the stronger feeling of loneliness while working from home among those who share their on-site office with more colleagues. It could also be assumed that those who share their on-site office with multiple colleagues are more likely to have the opportunity to receive support from their coworkers. The lack of support from coworkers is strongly related to feeling of loneliness (Jones, 1981; Wright, 2005). Those who share their on-site office with colleagues may miss this support more when working from home. Perhaps, people who work on-site in an office without colleagues have already developed remote strategies for regularly getting support from colleagues and can apply these strategies when working at home.

Given that very few vocational counselors predominantly did WFH but still worked in their office during most working days, the current study might underestimate feeling of loneliness and its associations with WFH. Replication of the study is needed in a sample that comprises more employees who mainly work from home because “the greatest impact on feelings of isolation appears to be telecommuting frequency. If people do not telecommute a lot, they will not be isolated” (Cooper and Kurland, 2002, p. 512). Moreover, feeling of loneliness might be different in employees who choose themselves to WFH compared to others who were forced into WFH during the COVID-19 pandemic (Kniffin et al., 2021).

In any case, it seems important to note the double-edged nature of WFH: WFH can increase resources like autonomy (Wood et al., 2021) and reduce distractibility and ultimately increase productivity and wellbeing, but this can also come along with costs such as feeling of loneliness and blurring of boundaries between work and home. However, it seems important that practical measures to prevent loneliness and to help people detach from work should be given high priority when WFH is implemented.

## Limitations

First, the main limitation arises from the cross-sectional data. Preferably, the WFH-related change in productivity is tested longitudinally. Moreover, changes in productivity and loneliness after frequent WFH may develop and change in time. So, even more than two measurement points are desirable. Second, bias from questionnaire responses as common source variance may have boosted correlations in this study (cf. Semmer et al., 2004). For instance, using client data on the helpfulness of vocational counseling as a productivity indicator could have helped prevent the common method variance (Semmer et al., 2004).

Third, we found that WFH was not related to a higher work–life balance. This finding may be due to a gender difference in total workload including work and private demands (Galliker et al., 2021b). At home, women are more likely to do WFH and may even increase their time spent on housework, increasing the work-life interference. The current study did not gather information about household work and other private demands and therefore could not test that potential gender-related effect. The *post hoc* regression analyses carried out separately for men and women showed that WFH was not associated with work-life balance in men and women. A test of the interaction between gender and WFH in association with work-life balance was not significant. Future studies on WFH should include household work and private demands.

Another important limitation refers to the unusual circumstances, in which the study took place. The COVID-19 pandemic entailed many restrictions regarding public and social life as well as working conditions. Shortly before the onset of data collection in October 2020, the Swiss Federal Council had increased protective measures against the then sharply rising infection rates. More specifically, it recommended to WFH whenever possible (Federal Council media release of October 18, 2020). Thus, it was not necessarily the own decision of an employee to work remotely. The question is therefore whether results can be generalized to non-pandemic working times. Finally, the study controlled work-related task stressors as predictors of productivity and other criterion variables, but did not assess work resources like social support (Chen et al., 2021) and individual factors that might contribute to successful work in WFH.

The study by Wang et al. (2021) showed that social support reduced feeling of loneliness only among workers who were high in self-discipline. It is assumed that occupational counseling psychologists may be relatively good in self-regulation skills like self-discipline, a hypothesis that should be investigated in future studies. Other individual characteristics related to better productivity in WFH might be future time orientation and proactivity (Chang et al., 2021). Given that very few vocational counselors predominantly did WFH but still worked in their office during most working days, the current study might underestimate feeling of loneliness and its associations with WFH.

## Conclusion

The COVID-19 pandemic increased WFH in vocational counselors. More frequent WFH was linked to higher productivity, lower distractibility, and higher job satisfaction. Vocational counselors who shared the office on-site with many colleagues experienced higher feeling of loneliness during WFH. The connection between WFH and work-life balance seems to depend on boundary management. Occupational health prevention should strengthen resources for boundary management in WFH.

## Data Availability Statement

The raw data supporting the conclusions of this article will be made available by the authors, without undue reservation.

## Ethics Statement

The studies involving human participants were reviewed and approved by Ethics committee of the University of Bern, Switzerland (12.01.2021, Ethics No. 2021-01-00001). The patients/participants provided their written informed consent to participate in this study.

## Author Contributions

AcE, SG, and AZ: conceptualization. AZ: data collection. SG and AcE: methodology and writing—original draft preparation. AZ, SG, NJ, PLM, AnE, and AcE: review and editing. All authors have read and agreed to the published version of the manuscript.

## Conflict of Interest

The authors declare that the research was conducted in the absence of any commercial or financial relationships that could be construed as a potential conflict of interest.

## Publisher’s Note

All claims expressed in this article are solely those of the authors and do not necessarily represent those of their affiliated organizations, or those of the publisher, the editors and the reviewers. Any product that may be evaluated in this article, or claim that may be made by its manufacturer, is not guaranteed or endorsed by the publisher.
